# A Comprehensive Comparison of Transmembrane Domains Reveals Organelle-Specific Properties

**DOI:** 10.1016/j.cell.2010.05.037

**Published:** 2010-07-09

**Authors:** Hayley J. Sharpe, Tim J. Stevens, Sean Munro

**Affiliations:** 1 MRC Laboratory of Molecular Biology, Hills Road, Cambridge CB2 0QH, UK; 2 Department of Biochemistry, University of Cambridge, Cambridge CB2 1GA, UK

**Keywords:** CELLBIO

## Abstract

The various membranes of eukaryotic cells differ in composition, but it is at present unclear if this results in differences in physical properties. The sequences of transmembrane domains (TMDs) of integral membrane proteins should reflect the physical properties of the bilayers in which they reside. We used large datasets from both fungi and vertebrates to perform a comprehensive comparison of the TMDs of proteins from different organelles. We find that TMDs are not generic but have organelle-specific properties with a dichotomy in TMD length between the early and late parts of the secretory pathway. In addition, TMDs from post-ER organelles show striking asymmetries in amino acid compositions across the bilayer that is linked to residue size and varies between organelles. The pervasive presence of organelle-specific features among the TMDs of a particular organelle has implications for TMD prediction, regulation of protein activity by location, and sorting of proteins and lipids in the secretory pathway.

## Graphical abstract

**Graphical Abstract:**

**Figure:**
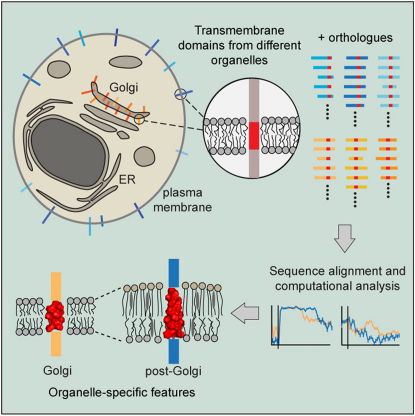

**Highlights:**

► Transmembrane domains (TMDs) vary in length and residue composition between organelles ► TMD lengths differ pre- versus post-Golgi but not between apical and basolateral surfaces ► The differences between TMDs are large enough to have value in predicting location ► Pervasive differences mean TMDs could collectively contribute to membrane properties

## Introduction

Integral membrane proteins are encoded by ∼30% of the genes in most genomes and perform numerous biological processes from signaling to transport (Almén et al., 2009; Stevens and Arkin, 2000). There are many indications that the activity of such proteins can be affected by physical properties of the lipid bilayer such as lipid order and hydrophobic thickness (Andersen and Koeppe, 2007; Bondar et al., 2009; Nyholm et al., 2007; Phillips et al., 2009). There is also considerable interest in the possibility that local differences in the physical properties of membranes could contribute to the lateral segregation of proteins during sorting or signaling (Bretscher and Munro, 1993; Dukhovny et al., 2009; Patterson et al., 2008; Ronchi et al., 2008; Simons and Ikonen, 1997). Determining the biological significance of such processes in eukaryotes is contingent on understanding the properties of the different bilayers of the cell. Organelle membranes vary in both their protein and lipid content, and even within one membrane the lipid composition of the two leaflets of the bilayer can be very different (van Meer et al., 2008). For instance, sterols and sphingolipids are scarce in the ER but abundant and asymmetrically distributed in the plasma membrane. These lipids differ from typical phospholipids in that sphingolipids are characterized by saturated acyl chains, and sterols by an inflexible core formed by four fused rings. In artificial liposomes the degree of acyl chain saturation and the levels of sterols affect such physical properties of the bilayer as thickness, order and viscosity (Brown and London, 1998). However, what effect they have at physiological levels in heterogeneous, protein-containing biological membranes is unclear.

Most integral membrane proteins contain α-helical transmembrane domains (TMDs) that span the hydrophobic core of the lipid bilayer (Killian and von Heijne, 2000; White and Wimley, 1999). The primary constraint on all TMDs that enter the secretory pathway is that they must partition out of the Sec61 translocon into the membrane of the ER during synthesis. TMDs are greatly enriched in aliphatic hydrophobic residues, and these residues promote partitioning out of the translocon (Hessa et al., 2005, 2007; Killian and von Heijne, 2000). However, the physical properties of the bilayer in which a protein will eventually reside should also impose constraints upon the sequence of its TMD. Previous studies comparing the TMDs of Golgi and plasma membrane proteins have suggested a difference in TMD length and hence bilayer thickness (Bretscher and Munro, 1993; Levine et al., 2000). However, the full significance of this finding for cellular organization is unclear as the analysis was based on only a small number of proteins and did not include other organelles. Indeed the conclusions have been called into question by attempts to measure bilayer thickness of different compartments (Mitra et al., 2004).

To obtain a clear picture of organelle-specific constraints on TMDs, we have made use of the recent increase in available genome sequences to perform a comprehensive comparison of a large number of membrane proteins with a single TMD from the major secretory organelles from both fungi and vertebrates. Our findings validate the previous suggestions of a difference in TMD length between Golgi and plasma membrane and extend this to reveal an apparent step-change in bilayer thickness that occurs in the secretory pathway at the trans side of the Golgi. We also find that the TMDs of proteins from post-ER organelles show striking variations in amino acid composition across the bilayer. This results in an asymmetry in residue composition that is linked to residue volume and correlates with changes in lipid asymmetry. Thus, eukaryotic TMDs are not a single type of entity but vary in a manner that implies that there are clear differences in the physical properties of the bilayers of the secretory pathway.

## Results

### Computational Analysis of Fungal and Vertebrate Transmembrane Sequences from Distinct Subcellular Locations

**Figure 1:**
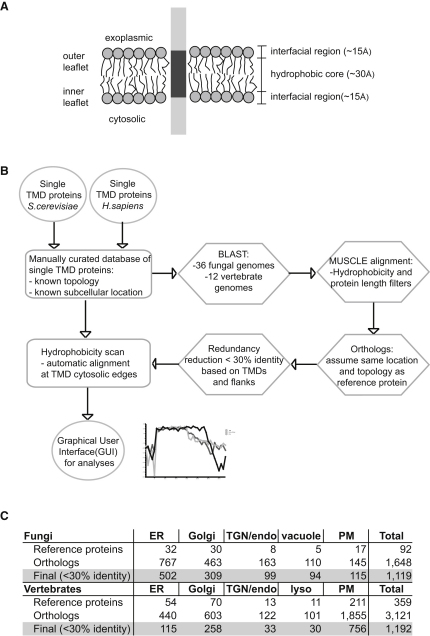
Overview of the Methodology for TMD Analysis (A) Schematic of a typical single-pass or bitopic protein embedded in a lipid bilayer. (B) Bitopic proteins of known topology and location from *S. cerevisiae* and *H. sapiens* were identified by literature and database searches. Orthologous proteins were identified using BLAST and aligned with the reference proteins. The starts of the TMDs were identified by a hydrophobicity scanning algorithm and used to align the TMDs at their cytosolic edges. (C) The number of proteins from the indicated organelles that were used in the analyses of TMDs (PM, plasma membrane). Redundancy reduction was such that TMDs and flanking sequences have <30% identity. Reference proteins are listed in Table S1 and Table S2. See also Figure S1.

To reliably compare TMDs that span different membranes, we curated a dataset of proteins with an experimentally determined topology and location and a single TMD (bitopic proteins, Figure 1A). Bitopic proteins represent ∼40% of all membrane proteins in eukaryotic genomes, and their TMDs are those likely to have the most residues exposed to the lipid bilayer (Almén et al., 2009; Krogh et al., 2001). We assembled datasets of all single TMD proteins from what are probably the best characterized eukaryotic genomes, *Saccharomyces cerevisiae* and *Homo sapiens*. We then used literature searches and cross-referencing between databases to identify those proteins with a known organelle of residence and topology (Table S1 and Table S2). For the Golgi apparatus we pooled all the proteins from the various cisternae of the Golgi stack into a single “Golgi” set, with a separate set for those proteins that cycle between the trans-Golgi network (TGN) and endosomes. Only a few mammalian Golgi proteins have been accurately located within the Golgi stack, but for yeast, where this is more easily done, we found that the proteins of the early part of the stack were strikingly similar in TMD properties to those from the later part of the stack (see below), indicating that this pooling probably does not mask significant complexity.

Selecting only those proteins with a known location and topology inevitably reduced the size of the datasets, and so to expand the number of sequences available for analysis, we used BLAST searches to collect the orthologous proteins from all other complete fungal and vertebrate genomes. The topology and subcellular location of orthologs were assumed to be the same as for the reference protein. Many of their functions are highly organelle specific, and a global comparison of protein localization in the distantly related yeasts *S. cerevisiae* and *Schizosaccharomyces pombe* found the subcellular distributions of orthologs to be very similar (Matsuyama et al., 2006). The inclusion of orthologs significantly expanded our datasets, but this would be of little value if the proteins were very similar to the reference sequence. Thus the proteins from each organelle set were redundancy reduced by using BLASTClust to cluster them based on sequence similarity in their TMD and flanking sequences, and then we removed any with greater than 30% identity over this region (Altschul et al., 1997). Figure 1B summarizes the strategy used, and the numbers of proteins used for the analysis are provided in Figure 1C.

### Alignment of TMDs Based on Their Cytosolic Ends

To compare the TMDs from different organelles, their sequences were aligned using the cytosolic ends of their hydrophobic cores. Initially, TMDs were located in the reference proteins using the TMHMM prediction algorithm (Krogh et al., 2001), and the orthologs were then aligned with the reference protein in order to assign their TMD positions. There is no established computational method for defining the ends of the part of a protein that spans the bilayer. Thus we implemented a scanning algorithm, which uses a sliding window and a threshold based on hydrophobicity. For this and subsequent analyses we used the hydrophobicity scale of Goldman, Engelman, and Steitz (GES) as it is designed for single-pass transmembrane helices and out-performs other scales in TMD prediction (Engelman et al., 1986; Koehler et al., 2009). However, to ensure that our findings were not dependent on this choice we also performed parallel analyses with the Wimley-White scale and the recently reported Biological scale from Hessa and coworkers, which is based on a completely different method (Hessa et al., 2007; Wimley and White, 1996). There is of course some flexibility in how charged residues are positioned at a bilayer interface, but by applying the same objective method to all organelles we should avoid bias in how TMD ends are assigned for the different datasets.

The scanning algorithm enabled us to align proteins from an organelle set at the position where a sharp change in hydropathy occurred, and the cytosolic end of the hydrophobic region was defined as position one. For all our analyses the hydrophobic spans were aligned with respect to their bilayer orientation, i.e. from the cytosolic side to the exoplasmic side (Figure 1A), rather than from N terminus to C terminus. We wanted to determine if residue preferences were influenced by position in the bilayer, which would be missed if all proteins (type I/III and type II) were simply analyzed from N to C terminus. In addition, the “positive-inside rule” indicates that the cytosolic flanking regions of TMDs are generally enriched in positively charged residues, thus allowing a clear definition of the cytosolic edges of hydrophobic spans (Nilsson et al., 2005).

### TMDs from Different Organelles Exhibit Compositional Differences

**Figure 2:**
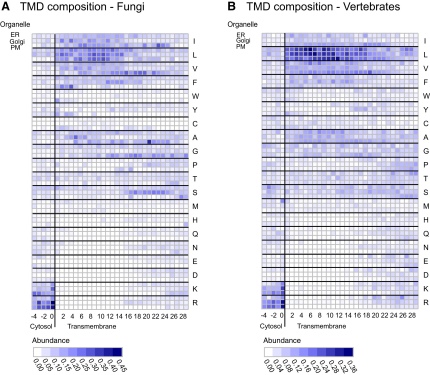
Positional Analysis of Amino Acid Composition of TMDs from Different Organelles in Fungi and Vertebrates (A and B) The position relative to the cytosolic edge of the TMDs is on the horizontal axes, and the amino acids and organelles are on the vertical axes. Amino acids are listed in order of decreasing hydrophobicity (Goldman-Engelman-Steitz [GES] scale [Engelman et al., 1986]). Normalized residue abundance is color-coded such that white represents zero and dark blue a maximum of one. The abundance of serines in the region following the lumenal end of Golgi TMDs probably reflects the fact that this part of many Golgi enzymes forms a flexible linker that tethers the catalytic domain to the membrane (Paulson and Colley, 1989). Graphical plots for individual residues can be generated at http://www.tmdsonline.org. See Table S3 and Table S4 for numerical values.

Using the aligned sets of proteins, the frequency of each amino acid at each position through the hydrophobic region was calculated and plotted as matrices for fungi and vertebrates (Figures 2A and 2B, numerical values in Table S3 and Table S4). The residue preferences typically show a cluster of basic residues on the cytosolic side, followed as expected by the run of mostly aliphatic hydrophobic residues that spans the hydrophobic core of the bilayer. However, the matrices also reveal striking compositional differences between, and along, the TMDs. For both fungi and vertebrates, the regions enriched in hydrophobic residues are shorter for the ER and Golgi proteins than for plasma membrane proteins, indicating a difference in TMD length. In addition, the different hydrophobic residues were not uniformly distributed through the hydrophobic TMD core. For example valine shows a clear enrichment in the exoplasmic side of the plasma membrane set in both vertebrates and fungi (Figure 2). To determine the extent and significance of such trends, we analyzed in more detail the changes in residue property and type through the bilayer.

### Hydrophobic Lengths of TMDs Differ along the Secretory Pathway in Fungi and Vertebrates

**Figure 3:**
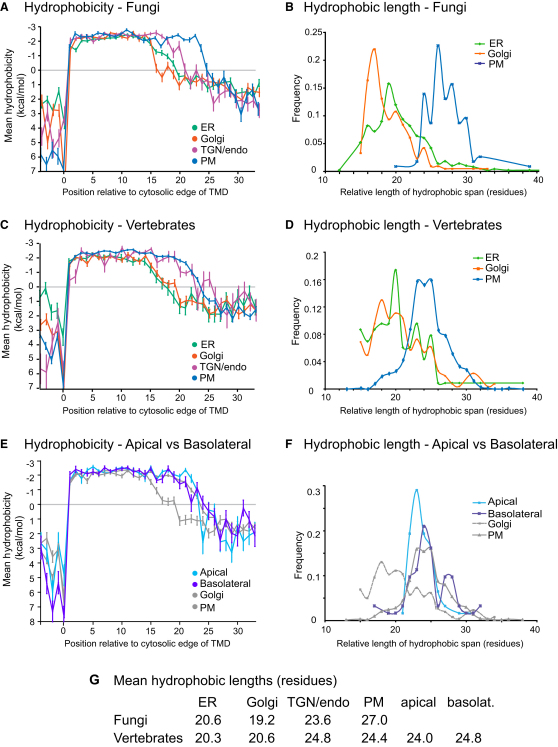
Positional Analysis of TMD Hydropathy from Different Organelles in Fungi and Vertebrates (A) The mean hydrophobicity (GES scale) of the residues at each position along the aligned TMDs relative to the cytosolic edge was plotted for the indicated protein sets from fungi. The hydrophobicity values represent the free energy for partitioning from water into a hydrophobic environment, and therefore negative values indicate a preference for the interior of a lipid bilayer. Bars show standard error of mean. (B) The distribution of TMD lengths for fungal organelles. The exoplasmic ends of the TMD were defined using the hydrophobicity scanning algorithm as for the cytosolic ends. (C and D) As for (A) and (B), but for vertebrate proteins. (E and F) As for (C) and (D), but for vertebrate proteins of the apical and basolateral domains of the plasma membrane. The Golgi and total plasma membrane plots from (C) and (D) are included for comparison. The 15 apical and 12 basolateral reference proteins are listed in Table S2. (G) The mean values for the TMD hydrophobic lengths of the indicated organelles shown in (B) and (D). For fungi, the differences between Golgi and TGN, Golgi and plasma membrane (PM), and TGN and PM are statistically significant (p < 10^−12^, two sample t tests), whereas for vertebrates this was the case for Golgi and TGN and Golgi and PM (p < 10^−10^) but not TGN and PM. See also Figure S2 for tests of robustness and significance of data.

**Figure S1:**
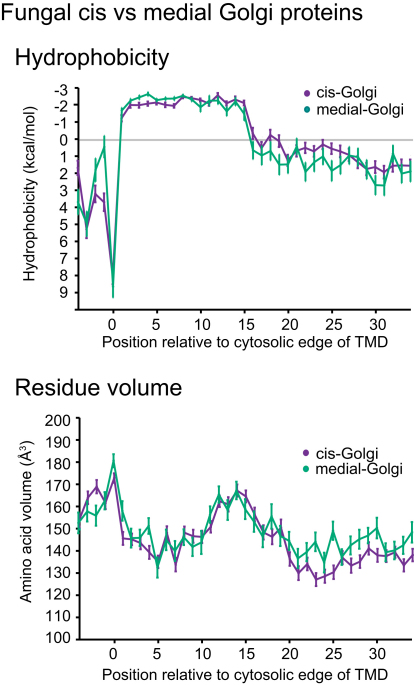
Properties Shared between TMDs from Early and Late Golgi, Related to Figure 1 Fungal proteins from the early (*cis*) and later (medial) parts of the Golgi data set were analyzed by plots of mean residue hydrophobicity and amino acid volume. Error bars indicate standard error of the mean.

**Figure S2:**
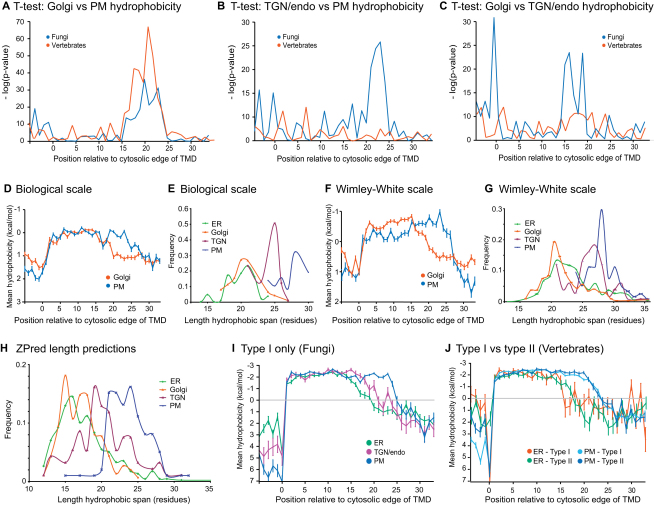
Analysis of the Significance and Robustness of Organelle-Specific Differences in TMD Hydropathy Plots, Related to Figure 3 (A, B, and C) Independent (two sample) t tests were used to compare the mean residue hydrophobicity of residues at positions along the TMDs of proteins from (A) the Golgi and plasma membrane (PM); (B) TGN/endosomes and plasma membrane (PM); and (C) the Golgi and TGN/endosomes. (D) Positional analysis of mean hydrophobicity of the Golgi and plasma membrane TMDs calculated using the Biological scale reported by Hessa and coworkers (Hessa et al., 2007). The Biological scale was also used to define cytosolic TMD edges and thus align the fungal TMDs from different organelles at their cytosolic ends. Error bars indicate standard error of the mean. (E) Distribution of apparent lengths of fungal TMDs calculated by using the Biological scale to also define both the cytosolic and exoplasmic TMD edges. (F and G) As for (D) and (E) except that the Wimley-White hydrophobicity scale was used to define the TMD edges and calculate the mean hydropathy at each position (Wimley and White, 1996). (H) Distribution of lengths of TMDs obtained from the output of the TMD prediction program Zpred2 (Papaloukas et al., 2008). TMDs with 10 flanking residues on either side were used as the input for Zpred2 and the output was parsed to give a TMD length for each protein. In all cases a similar trend is seen: ER and Golgi TMDs are generally shorter than plasma membrane TMDs, with the TGN/endosome set being intermediate. (I and J) Analysis of mean TMD hydropathy of proteins of different topology using the GES scale. Results are shown for type I fungal proteins from the ER, TGN/endosomes and plasma membrane (PM) datasets (I), and for vertebrate type I and type II proteins from the ER and plasma membrane datasets (J). Error bars indicate standard error of the mean.

To quantify trends in hydropathy, the mean hydrophobicity over all the sequences in each dataset was plotted relative to residue position. As noted above, the hydropathy plots for the fungal proteins from the early Golgi and late Golgi were found to be very similar, and so the datasets were combined to form a “Golgi” set (Figure S1 available online). For both fungi and vertebrates, the plasma membrane TMDs were on average hydrophobic for a greater length than those of the ER and Golgi (Figures 3A and 3B). For fungi the hydrophobicity values of the Golgi and plasma membrane TMDs were highly significantly different between positions 16 and 24 (p < 1 × 10^−10^ from two-sample independent t test, Figure S2A). For vertebrates, the difference between Golgi and plasma membrane TMDs was highly significant for positions 17 to 23 (Figure S2A).

To determine the prevalence of this difference in length within the datasets, we used the scanning algorithm described above to also define the exoplasmic ends of the TMDs and thus obtain a measure of the hydrophobic length for all of the TMDs. Distribution plots of TMD length show clearly distinguishable profiles for Golgi versus plasma membrane proteins (Figures 3C and 3D), with mean values that are highly significantly different (Figure 3E). As described above, the definition of TMD ends and the analyses of hydrophobic lengths were also performed using the Wimley-White and Biological hydrophobicity scales to avoid bias arising from using one particular hydrophobicity scale (Hessa et al., 2007; Wimley and White, 1996). The plots of TMD hydropathy and TMD length distribution determined using these differently derived scales show very similar trends to those obtained with the GES scale (Figures S2D–S2G). We also examined the distribution of TMD lengths predicted for the proteins by TMD prediction program Zpred2 (Papaloukas et al., 2008) and again found similar trends (Figure S2H).

Overall, plasma membrane TMDs have a greater hydrophobic length than those TMDs that span Golgi membranes, and this difference is conserved between fungi and vertebrates. However, the plots also reveal some differences between fungi and vertebrates. In fungi, the TGN/endosomal TMDs have a mean hydrophobic length intermediate between those of the Golgi and plasma membrane TMDs. However, in vertebrates, the TGN/endosomal TMDs appear to be of similar lengths to those of the plasma membrane. Indeed, the mean hydrophobicity values of the two sets of vertebrate proteins are not very significantly different in the region where the TMDs come to their end (residues 16–24), whereas for the fungal proteins there is a highly significant difference between the plasma membrane and TGN/endosomal TMDs at positions 21–24 (p < 1 × 10^−10^, Figure S2C).

There have been suggestions that interactions with lipids could contribute to the sorting of membrane proteins to the apical surface in polarized epithelia (Simons and van Meer, 1988). We identified 15 apical and 12 basolateral reference proteins with a single TMD that were expanded to sets of 62 of each after adding orthologs and redundancy reduction to <30% identity (Table S2). However, the hydrophobic plots of the two sets are similar to each other and to the total plasma membrane set (Figure 3E), and the apical TMDs appear no longer than those of the basolateral surface (Figure 3F).

### TMD Lengths Vary along the Secretory Pathway Irrespective of Type I versus Type II Topology

We noted that the Golgi datasets from both species groups consist only of proteins with a type II topology (N terminus in the cytosol). Conversely, the plasma membrane proteins from the fungal set all have a type I topology. It has been reported that topology has little influence on the sequence of TMDs in terms of partitioning out of the translocon (Lundin et al., 2008). Nonetheless, it seemed important to address the possibility that the trends observed here relate to differences in topology rather than location. Thus, we divided the organelle sets on the basis of topology. In fungi, there are type I proteins in the ER and TGN/endosomes sets in addition to the plasma membrane. The hydropathy plot in Figure S2I demonstrates that the TMDs of type I proteins from the plasma membrane are longer than those of type I proteins from the TGN/endosomes and ER sets, as observed for the combined topologies. For vertebrates, both type I and II proteins are present in the plasma membrane and ER datasets. The hydrophobicity plot in Figure S2J demonstrates that the plasma membrane proteins have longer average hydrophobic regions than ER proteins irrespective of whether type I or type II proteins are compared. Thus the trends we observe in hydrophobic length appear related to subcellular location rather than topology.

### Hydrophobic Residues Are Distributed Asymmetrically in Plasma Membrane and Golgi TMDs

**Figure 4:**
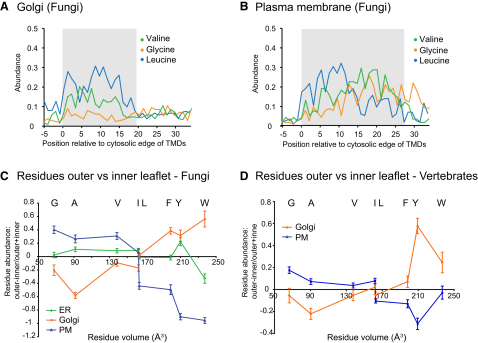
Analysis of the Compositional Asymmetry of TMDs from Different Organelles of Fungi and Vertebrates (A and B) Analysis of the abundance of valine, glycine, and leucine along the TMDs from Golgi and plasma membrane proteins of fungi. Shaded areas represent the mean length of the hydrophobic regions for each protein set (Figure 3G). (C and D) Analysis of amino acid asymmetry in ER, Golgi, and plasma membrane (PM) TMDs from fungi and in Golgi and plasma membrane TMDs from vertebrates. The abundance of each residue in the “inner” leaflet was subtracted from the abundance in the “outer” leaflet and divided by the total abundance to give a ratio of leaflet preference (0 = no preference). Leaflet position was defined by dividing the mean hydrophobic length for each organelle into two equal parts, and values for the different residues are plotted along the x axis according to residue volume. Error bars represent the standard error of the mean.

Although the hydrophobic cores of the TMDs from the various organelles differ in length, they all have similar hydropathy values that do not vary greatly along the length of this core. However, the residue frequency plots above suggest that the abundance of individual hydrophobic residues changes along the length of the TMDs (Figure 2). The residues valine, glycine, and leucine are uniformly distributed through the fungal Golgi TMDs, but all are asymmetrically distributed in plasma membrane TMDs, with valine and glycine being favored in more exoplasmic positions, whereas leucine shows the opposite trend (Figures 4A and 4B). To quantify further the degree of residue asymmetry, the relative lengths of each TMD in an organelle set were calculated as above and used to define the halves of the TMD corresponding to the inner and outer leaflets of the membrane. The abundance of each amino acid in the “inner” leaflet was subtracted from the abundance in the “outer” leaflet and divided by the total abundance to give a ratio for the leaflet preference. The mean ratios for each hydrophobic residue in each organelle set are shown in Figures 4C (fungi) and 4D (vertebrates). The values are plotted against the volume of each amino acid residue (Pontius et al., 1996). For fungal plasma membrane proteins, the overall trend is for the outer leaflet half of the TMD to have an increase in smaller residues and decrease in larger residues, with the opposite trend for the Golgi proteins, whereas ER TMDs show no difference in relative abundance of hydrophobic residues between the leaflets. For vertebrates, a comparison of Golgi and plasma membrane asymmetry shows a similar trend to that seen in fungi, albeit smaller in scale. Overall, these results suggest that the constraints on amino acid composition of TMDs differ between the two leaflets of the bilayer.

### TMD Compositions Appear Constrained by Residue Volume

**Figure 5:**
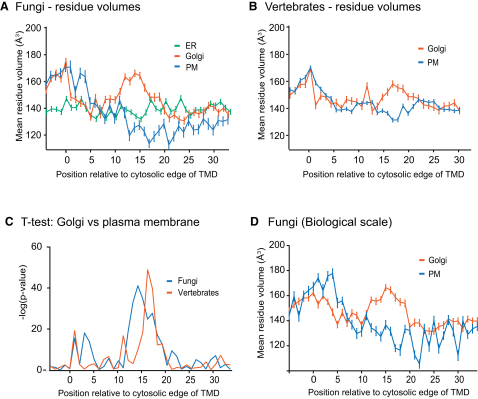
Positional Analysis of Amino Acid Volume from Different Organelles in Fungi and Vertebrates (A and B) The mean values for residue volume (Pontius et al., 1996), at each position along the TMDs from fungi and vertebrates. Error bars indicate standard error of the mean. (C) Independent (two sample) t tests were applied at positions along the TMDs to assess the significance of differences between the mean values of amino acid volumes for Golgi and plasma membrane proteins shown in (A) and (B). (D) The Biological scale of Hessa and coworkers was used to define cytosolic TMD edges and thus align the TMDs from different organelles at their cytosolic ends (Hessa et al., 2005). This alignment was then used for analysis of amino acid volume along the fungal Golgi and plasma membrane TMDs. Error bars indicate standard error of the mean. See also Figure S3.

The asymmetry described above appears to correlate to residue volume, and so we calculated the mean residue volume at positions along the TMDs. Figure 5A shows that Golgi and plasma membrane TMDs from fungi have similar profiles of residue volume in the halves of their TMD closest to the cytosol (positions 1–11 from the cytosolic side). However, in the exoplasmic portion of the TMDs, there is a bifurcation after residue 11; the plasma membrane TMDs have smaller mean volumes and the Golgi larger ones, with these differences being seen over most of the exoplasmic part of the TMDs. A similar trend is seen for the vertebrate proteins, with mean residue volumes similar in the cytosolic half and then splitting after position 12, with the larger amino acids for the Golgi TMDs and smaller amino acids in the plasma membrane TMDs (Figure 5B). The differences in amino acid volume are highly statistically significant for positions 12–19 in fungi and positions 14–19 in vertebrates (p < 1 × 10^−10^ for both, Figure 5C). In addition, very similar differences are observed if the TMD ends are defined with the Biological scale instead of the GES scale (Figure 5D). This indicates that there is an increase in average residue volume in the outer leaflet portion of TMDs from Golgi proteins and a reduction in volume for this part of plasma membrane TMDs.

### Plasma Membrane TMDs Do Not Display a “Size Moment”

**Figure S3:**
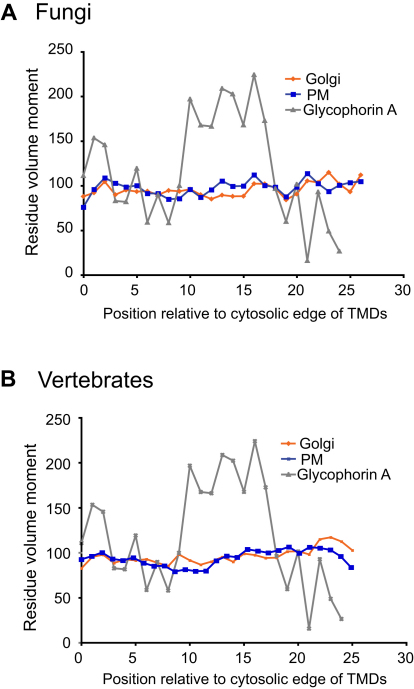
Positional Analysis of Residue Size Moment, Related to Figure 5 (A and B) The size moment at each position of a protein was defined as being the sum of the vector of the volume of that residue and of the vectors of the volumes of the six residues on either side (i.e., a window of seven residues which approximates to two turns of an α helix—see Experimental Procedures). This moment was calculated at each position for each protein and mean values at each position determined for all the proteins in the same dataset. Glycophorin A dimerizes via a GXXXG motif in its TMD and serves as a positive control. The different datasets from the different organelles do not show large differences in size moment, and hence are not differentially enriched in GXXXG-like dimerization motifs.

One possible explanation for the reduction in average residue volume in the exoplasmic side of the plasma membrane TMDs is that we were detecting a relative enrichment of GXXXG-like oligomerization motifs. This motif aligns two glycines or other small residues on one face of the helix, and these allow the TMDs to pack closely and dimerize via their backbones (Russ and Engelman, 2000). In order to test if this was the case, we quantified the helical size bias of the TMDs in the different datasets. Residue volume was treated as a vector from the helix, and the values summed for two turns (i.e., seven successive residues) to give a “size moment.” If one side of the helix is flattened, i.e., has more small residues than the opposing side, then the size moment will be higher over that region. Glycophorin A has a GXXXG motif within its TMD and was used as a positive control (Russ and Engelman, 2000). The plasma membrane TMD sets do not show a large peak in size moment in their exoplasmic positions such as that seen for glycophorin A (Figures S3A and S3B). This implies that the exoplasmic parts of the plasma membrane datasets are not substantially enriched in flat dimerization motifs and indicates that the increased proportion of smaller residues instead reflects a difference in overall residue composition all round the transmembrane helix.

### An Artificial Neural Network Can Classify Subcellular Location Based on TMD Sequence

**Figure 6:**
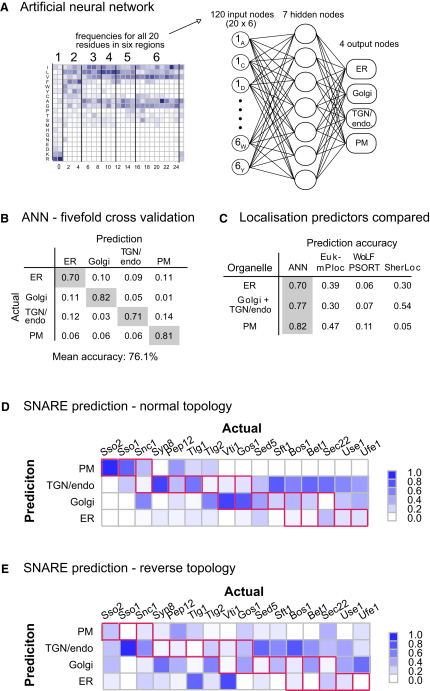
An Artificial Neural Network Classifier of Subcellular Location Based on TMD Sequence (A) Overview of the neural network used for classifying proteins. The compositions of six regions along the TMDs from each fungal organelle set were encoded into input vectors to train the network. (B) Test of the accuracy of the ability of the neural network to predict localization. Performance was assessed using a 5-fold “leave-one-out” cross-validation in which groups of proteins were removed from the training set and then used to test the network trained with the remaining proteins. The predicted location was that with the highest score, with a mean accuracy calculated over all proteins in each set. (C) A comparison of predictive accuracy of the network (ANN) to that of existing subcellular localization prediction methods when applied to the *S. cerevisiae* reference proteins. (D and E) Prediction of SNARE localization using the neural network trained on TMD regions. The SNAREs from *S. cerevisiae* and 36 other fungi were examined with the network trained on the datasets that do not include the SNAREs, and the frequencies of predictions were normalized and plotted in a matrix against subcellular locations. Red boxes indicate the experimentally determined localizations of the SNAREs. SNARE TMD sequences were reversed prior to analysis in (E).

To evaluate further the scale of organelle-specific heterogeneity among TMDs, we tested whether the differences are sufficiently great to have predictive value. An artificial neural network was implemented with the aim of classifying proteins into organelles using the amino acid composition of delineated regions through the TMDs (Figure 6A). To avoid bias arising from differences in dataset size, proteins were randomly removed from the redundancy-reduced sets of fungal proteins such that each organelle was represented by the same number of proteins (n = 99). The neural network was then trained on these fungal proteins from the ER, Golgi, TGN/endosomes, and plasma membrane. Networks were tested by cross-validation in which groups of proteins were removed from the training set and then used to test the network trained with the remaining proteins. Using a 5-fold cross-validation, the network correctly classified over 70% of proteins from ER, early Golgi, TGN/endosomes, and plasma membrane (Figure 6B).

We compared this outcome to that obtained with widely used algorithms for predicting subcellular localization. The most recent methods for location prediction are based on a combination of text-mining and ab initio sequence-based methods. We thus challenged three of the major location predictors (WoLF PSORT, SherLoc, Euk-mPLoc) with the complete sequences of the *S. cerevisiae* proteins from our datasets (Chou and Shen, 2007; Horton et al., 2007; Shatkay et al., 2007). The neural network, using only the sequence of the TMDs, outperformed all three predictors using the complete protein sequences when classifying bitopic proteins between ER, Golgi, and plasma membrane (Figure 6C). This suggests that incorporation of analysis of TMD sequences could improve the accuracy of current methods for predicting subcellular localization.

### SNARE TMDs Exhibit Organelle-Specific Trends in Composition

To seek further evidence for organelle-specific constraints on TMDs, we used the neural network to examine the proteins of SNARE family that mediate fusion between vesicles and organelles (Jahn and Scheller, 2006). Most SNAREs have a single TMD, and these all have the same type II topology. Individual SNAREs all perform the same general fusogenic role but are located to different organelles within the exocytic and endocytic pathways. They were not included in the datasets analyzed above, and so we tested whether the neural network could detect organelle-specific differences in the TMDs of the SNARE proteins. Predictions were performed on the fungal orthologs of all of the SNAREs from *S. cerevisiae* (Figure 6D). Overall, the outcome was far from random with SNAREs from the early secretory pathway predicted to be ER or Golgi, and plasma membrane and endosomal SNAREs predicted to be TGN/endosomes or plasma membrane. The accuracy of prediction is less than that obtained with the datasets examined above (50% correct rather than >70%), which may reflect the multitude of factors involved in the recycling and localization of SNAREs, and the TMDs potentially having a role in SNARE function (Stein et al., 2009). However, when the sequences of the SNAREs were reversed, and hence the orientation of their TMDs with respect to the bilayer, there was no particular trend or accuracy in the prediction (22% correct, Figure 6E). Thus, despite the SNAREs all sharing a common general function, there are constraints imposed on the sequences of SNARE TMDs that are shared with the TMDs of unrelated proteins from the same organelle, and the asymmetry of these constraints is a major feature detected by the neural network.

## Discussion

**Figure 7:**
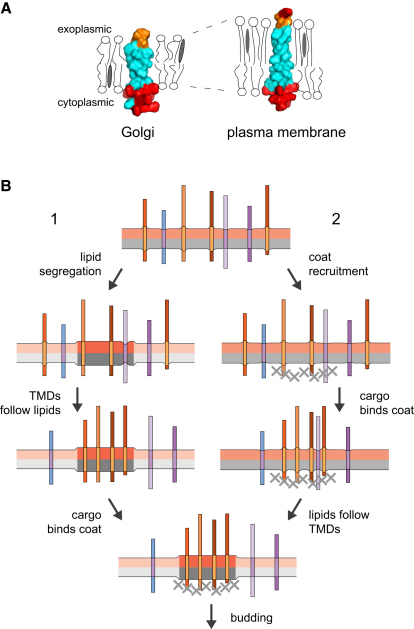
Organelle-Specific TMDs and Their Relationship with the Lipid Bilayer (A) Consensus TMDs from the fungal Golgi and plasma membrane datasets based on the most abundant residue at each position. Residues were modeled on an α helix using PyMOL. Hydrophobic residues (AGILVFWY) are colored cyan, polar residues (HNQST) orange, and basic residues (KR) red. The representation of the bilayer assumes that the plasma membrane is thicker and has a higher content of saturated lipids and sterols in the outer leaflet than do Golgi membranes. (B) Sorting of proteins sharing distinct TMD properties could either be driven by lipid sorting or could drive lipid sorting. For example, a domain of thicker, more ordered lipids could attract proteins with longer TMDs, and these could then attract a coat (1). Alternatively, if the cargo proteins for a particular class of vesicle have longer TMDs than the resident proteins, then their collection by coat into a forming transport carrier could affect the lipid composition around them, which would sort lipids and exclude residents with shorter TMDs (2). Either system could alternatively act on short TMDs if they were collected into vesicle by coats or segregated into thinner domains. See also Figure S4.

The analysis described here is, to the best of our knowledge, the first report of a comprehensive comparison of TMDs from all the major compartments of the eukaryotic secretory pathway. We find overwhelming evidence that there is not a “generic” type of TMD shared by eukaryotic membrane proteins. There are, of course, protein-specific constraints on TMD sequences imposed by the interactions and function of a particular protein. However, it appears that TMDs also vary depending on their organelle of residence in both length and composition. The structural consequences of these compositional differences can be illustrated by modeling the “consensus” TMDs for the fungal Golgi and plasma membrane (Figure 7A). These organelle-specific trends have obvious implications for improving the prediction of TMD presence and topology, as TMD features recognized by prediction algorithms will, in part, reflect the localization of the membrane proteins used to train the algorithm.

Our observations also have implications for how and why the different bilayers of the cell vary in their physical properties. The TMDs from the plasma membrane proteins of both fungi and vertebrates are longer than those from the proteins of internal membranes, even though the two sets of plasma membrane proteins are otherwise unrelated by sequence or function. This length difference was suggested by previous analyses of much smaller datasets from the plasma membrane and the Golgi (Bretscher and Munro, 1993; Levine et al., 2000) but is unequivocally validated by these much larger datasets. In addition, the analysis has now been extended to all of the secretory pathways of both vertebrates and fungi, revealing that TMD lengths are similarly short in both the ER and Golgi and then increase in compartments beyond the Golgi stack. This difference could reflect a shared tendency for post-Golgi TMDs to tilt in the bilayer of their organelle of residence, but this seems highly implausible, especially as the increased levels of order-inducing lipids in post-Golgi membranes would be expected to discourage tilting (see below). Thus the simplest explanation of the difference in TMD length is that for both fungi and vertebrates the plasma membrane is thicker than the membranes of the ER and Golgi. The length of an α helix increases by 1.5 Å per residue, and so these differences in TMD length would equate to an increase in bilayer thickness of ∼12 Å (42%) from Golgi to plasma membrane in fungi and ∼6 Å for vertebrates.

**Figure S4:**
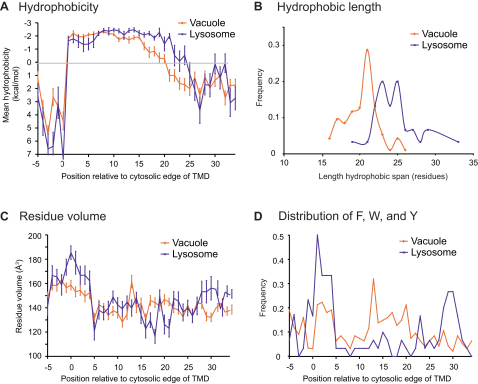
A Comparison of the TMDs from the Fungal Vacuole and Vertebrate Lysosome, Related to Figure 7 (A) Positional analysis of mean hydrophobicity relative to the cytosolic ends of TMDs. (B) Distribution of relative TMD lengths. (C) Positional analysis of amino acid volume. (D) Abundance of aromatic residues (phenylalanine, tryptophan and tyrosine) along TMDs. Error bars in (A) and (C) indicate the standard error of the mean.

Although the trend for longer TMDs in post-Golgi compartments is broadly similar in fungi and vertebrates, there also appear to be some differences. The TMD lengths imply that the fungal plasma membrane is even thicker than that of vertebrates, and also the membranes of the TGN/endosomal system are similar in thickness to the plasma membrane in vertebrates, but in fungi their thickness is intermediate between those of the Golgi and plasma membrane. The TGN/endosomal route is followed by proteins taken in from the plasma membrane or traveling from the Golgi to the vacuole or lysosome (Bonifacino and Traub, 2003; Bowers and Stevens, 2005). We did not include these lytic compartments in the analysis above because only a few bitopic proteins are known for each. However, when the methods used above are applied to these small datasets, the vertebrate lysosomal proteins appear similar to plasma membrane proteins, with longer TMDs and a preference for smaller residues in the exoplasmic half of the bilayer (Figure S4). In contrast, the fungal vacuolar proteins have shorter TMD lengths and an increased abundance of bulky aromatic residues compared to lysosomal TMDs (Figure S4). These differences cannot be viewed as definitive given the small numbers of reference proteins, but they are at least consistent with all post-Golgi membranes in vertebrates being equally thickened compared to the Golgi and ER, whereas in fungi the plasma membrane is particularly thick and the other post-Golgi membranes are intermediate in thickness compared to the Golgi.

The thickness of a fluid lipid bilayer has been shown to depend on acyl chain length and the presence of lipids such as sterols or sphingolipids (Brown and London, 1998; Lewis and Engelman, 1983). Sterols are rigid and sphingolipids have saturated acyl chains, and so both increase acyl chain order and thus thicken the bilayer and reduce permeability to solutes. The plasma membranes of fungi and mammals are enriched in sterols and sphingolipids compared to the ER and Golgi, which would be consistent with an increase in bilayer thickness (Holthuis et al., 2001). Sphingolipids are synthesized in the exoplasmic leaflet of the trans-Golgi from where they move, via mechanisms that are not understood, up a concentration gradient into post-Golgi compartments (Holthuis and Levine, 2005; Klemm et al., 2009; Tafesse et al., 2006; van Meer, 1989). The vacuole and endosomes of *S. cerevisiae* have relatively low levels of sterols and sphingolipids compared to the fungal plasma membrane or vertebrate lysosomes, which would be consistent with the apparent differences in the bilayer thickness between these organelles (Klemm et al., 2009; Schneiter et al., 1999).

In contrast, when we compared the TMDs of proteins that reside in the apical or basolateral domains of epithelial cells, we did not find a clear difference in hydrophobic length or trends in residue volume (Figure 3 and data not shown). There have been suggestions that TMD:lipid interactions could contribute to sorting of proteins to the apical surface (Simons and van Meer, 1988), but we are not aware of any previous report of a comparison of the TMDs from the two sets of proteins. The lack of apparent difference in TMD length may reflect the relatively small number of reference proteins, and indeed Mitra and coworkers have used low-angle X-ray scattering to measure the thickness of membranes of polarized hepatocytes and reported that the apical membrane was 3–5 Å thicker than the Golgi and ER, but the basolateral membrane was, if anything, thinner (Mitra et al., 2004). However, it should be noted that although X-ray scattering is an interesting approach, the method requires that organelles are isolated from cells, treated with carbonate to rupture them, and then treated for several hours with protease. This could perturb aspects of the bilayers and so may not have provided a definitive measure of in vivo properties. Moreover, the protocol used to isolate basolateral membranes removes apical membranes but not all others, with inner mitochondrial membranes alone constituting 22% of the basolateral fraction (Meier et al., 1984). It should also be noted that whereas glycolipids are ∼2-fold more concentrated on the apical surface of many epithelia, the other order-inducing lipids cholesterol and sphingomyelin can be equally distributed, and sphingomyelin even concentrated at the basolateral surface in some cell types (Brasitus and Schachter, 1980; Simons and van Meer, 1988; van IJzendoorn et al., 1997). Further work is clearly needed to understand the different properties of the apical and basolateral surfaces, but at present it seems possible that the major difference in bilayer thickness in epithelial cells could occur between pre- and post-Golgi compartments rather than between apical and basolateral domains.

In addition to variations in TMD length, we also found an asymmetry in the distribution of residue volume in the plasma membrane TMDs. Extrapolating from studies of bilayer permeability, small and more compact side chains would be expected to fit better into a bilayer that has well-ordered lipid acyl chains (Mathai et al., 2008; Mitragotri et al., 1999). This implies that there is an asymmetry in the state of lipid order in the plasma membrane. Such an asymmetry is more easily accounted for by lipids such as sterols and sphingolipids, which are enriched in one leaflet, than by proteins that span both leaflets. This suggests that lipids contribute, at least in part, to differences in bilayer order between organelles or subdomains. Indeed TMD asymmetry may explain why plasma membrane proteins show a surprising exclusion from “plasma membrane-like” lipid domains in liposomes (Bacia et al., 2004), as liposomes are symmetric and so the residues of the TMD adapted to the cytosolic leaflet would be exposed to a lipid organization that is only experienced in vivo by the outer leaflet residues.

The results of our analysis strongly imply that the different bilayers of eukaryotic cells have different physical properties, and these differences seem likely to be, at least in part, imposed by differences in lipid composition. Changes in membrane properties would provide an indication of location that could be used to control the activity of proteins such as channels and transporters as they move through the secretory pathway. However, a striking aspect of the data is how pervasive the differences between TMDs are in the large datasets that we have examined, implying that the TMDs of many of the proteins in a particular compartment share organelle-specific properties. This is perhaps clearest for TMD length in the case of fungi (Figure 3C), but even for vertebrates 92% of the plasma membrane TMDs are longer than the mean length for the Golgi and ER. Previous theoretical and experimental work has suggested that integral membrane proteins can influence the organization of the lipids that surround them (Andersen and Koeppe, 2007; Mitra et al., 2004; Mouritsen and Bloom, 1993). In addition, a quantitative analysis of the composition of synaptic vesicles revealed that TMDs account for ∼20% of the area of the membrane, indicating that most lipids are close to proteins, and this very high protein density is unlikely to be unique to this particular membrane (Frick et al., 2007; Takamori et al., 2006). If many of the proteins in the same compartment or forming vesicle share TMD shapes then they could contribute to bilayer properties, and in particular to thickness, if they are at a high enough concentration. Protein clustering in forming vesicles could thus cause local changes in bilayer physical properties, which could result in lipid sorting, especially at the late Golgi where sphingolipids are synthesized and a major transition in bilayer thickness seems to occur (Figure 7B). This means that the answer to the long-standing question of how cells sort lipids to different destinations could be that it is an emergent property of the traffic of membrane proteins that are at a high density and share organelle-specific TMD properties. This need not exclude the resulting protein/lipid microdomains attracting further cargo or excluding residents based on physical properties alone. Determining the relative contributions of proteins and lipids to each other's sorting is likely to be a key issue for future studies of the biogenesis of eukaryotic membranes.

Further work will be required to investigate these issues in detail, but irrespective of the outcome of such studies, our analysis clearly shows that eukaryotic TMDs are not a generic entity that is varied solely for protein-specific functions. Rather, TMD sequences are optimized for insertion, function, and also the variable and asymmetric physical properties of their bilayers of residence.

## Experimental Procedures

Full methods and associated references are in the Extended Experimental Procedures online. In summary, proteins with a single TMD from *S. cerevisiae* and *H. sapiens* were collated from databases. Those with a known location and topology were identified from the literature (Table S1 and Table S2), and their TMDs located with the prediction program TMHMM (Krogh et al., 2001). Orthologs from a further 36 fungi or 12 vertebrates were identified by BLAST searching of RefSeq genomes, and the TMDs in the orthologs identified by aligning them to the references sequences.

The cytosolic and exoplasmic edges of the TMDs were defined as the point at which the residue hydropathy in a small window sliding out from the middle of the TMD fell below a fixed threshold. For analysis of residue properties all the TMDs were aligned at their cytosolic edges. For type II proteins, residues were thus analyzed starting from the N-terminal end of their TMDs, and for type I and III from the C-terminal end. The resulting datasets were analyzed using custom software with a graphical user interface, and plots of residue properties or abundance can be generated at http://www.tmdsonline.org.

**Extended Experimental Procedures**

**Sequence Collation**

The proteome sequences of the fungi *S. cerevisiae, A. capsulatus, A. clavatus, A. fumigatus, A. nidulans, A. niger, A. oryzae, A. terreus, B. fuckeliana, C. albicans, C. cinerea, C. glabrata, C. globosum, C. immitis, C. neoformans, D. hansenii, E. gossypii, G. zeae, K. lactis, K. waltii, L. bicolor, L. elongisporus, M. grisea, M. globosa, N. fischeri, N. crassa, P. anserina, P. guillermondii, P. nodorum, P. stipitis, S. japonicus, S. kluyveri, S. pombe, S. sclerotorium, U. maydis, V. polyspora, Y. lipolytica,* and the vertebrates *H. sapiens, B. taurus, C. familiaris, D. rerio, E. cabullus, G.gallus, M. domestica, M. mulatta, M. musculus, O. anatinus, R. norvegicus, S. scrofa,* and *T.guttata* were downloaded from RefSeq (Pruitt et al., 2007). Single-pass proteins from *S. cerevisiae* and *H. sapiens* with experimentally determined topologies and locations were identified from literature and database searches (*Saccharomyces* Genome, TopDB and LOCATE databases (Sprenger et al., 2008; Tusnády et al., 2008)), and grouped by subcellular location (Table S1 and Table S2).

**Ortholog Identification**

Orthologs of each of the single-pass proteins in Table S1 and Table S2 were identified using a BLAST (Basic local alignment search tool) based algorithm (Altschul et al., 1990). For *S. cerevisiae* proteins the searches were performed against the 36 fungal genomes above. For *H. sapiens* searches were performed against the 12 vertebrate genomes listed above. The cut-off stringency for BLAST was E = 10^−10^. For each protein the best hit from each species was collected if present. Relative TMD positions were obtained by aligning the orthologs to the reference proteins using MUSCLE (Edgar, 2004). Orthologs were filtered for deviation in expected protein length (±100 residues) and TMD hydrophobicity (window size 10, average threshold 0.95 kcal/mol), and duplicated proteins were removed.

**Redundancy Reduction**

To ensure that the analysis was not biased by the presence of closely related sequences, the BLASTClust option of the BLAST distribution was used to cluster sequences at 30% identity. The clustering was performed on sequences corresponding to the hydrophobic core of the TMD and 10 residues of flanking sequence from either side. For each organelle, one protein from each cluster was selected at random for the analysis, ensuring that no two proteins had greater than 30% identity in their TMD regions.

**Transmembrane Domain End Definition**

A hydrophobicity scanning algorithm was implemented to identify the point where a sharp change in hydropathy occurs in sequences known to have a TMD. The approximate TMD edges from TMHMM were used as guides (Emanuelsson et al., 2007). The edges were indented by 4 amino acids at one end or the other and then a window of five residues centered on the measured residue was scanned back toward the TMD end. The Goldman-Engelman-Steitz (GES) hydrophobicity scale was used unless stated (Engelman et al., 1986). Ends were defined by an average hydropathy across the window of more than −0.94 kcal/mol or by an individual residue with a hydropathy of more than 8.0 kcal/mol (D, E, K, or R). For comparison the scanning was also performed using the Biological and Wimley-White scales (Hessa et al., 2007; Wimley and White, 1996). The window threshold was the median value of each scale (0.20 kcal/mol for Biological or −0.50 kcal/mol for Wimley-White, and the individual residue threshold corresponded to the second most hydrophilic value (2.70 kcal/mol for Biological (K, D), or 3.60 kcal/mol for Wimley-White (D, E)). Altering these parameters such that the threshold was set to zero, or the individual residue criteria removed, did not substantially affect the plot profiles or conclusions for any scale (data not shown).

For analysis with ZPRED, a stand-alone version of Zpred2 was obtained from Arne Elofsson (Stockholm University) (Papaloukas et al., 2008). To obtain TMD lengths, the number of residues predicted to be within 15 Å of the membrane center was calculated. The analysis could not be performed on whole protein sequences, as the software cannot distinguish signal peptides from TMDs. Instead, FASTA files were created of the TMDs (as calculated above), with 10 flanking residues on either side to eliminate the change of error from use of the GES scale for TMD end definitions.

**Analysis of Amino Acid Composition, Hydrophobicity, and Residue Volume**

For each position relative to the aligned cytosolic edge in a protein set, the frequency of each amino acid was calculated and normalized to one. The mean hydrophobicity (kcal/mol, GES Scale (Engelman et al., 1986), Biological scale (Hessa et al., 2007) or Wimley-White (Wimley and White, 1996)) and amino acid volumes (Å^3^, (Pontius et al., 1996)) for each organelle set were calculated for positions along the TMD. These data were then plotted as matrices, bar charts or line graphs within a custom-written graphical user interface in Python. The interface was built using Python graphical libraries developed as part of the CCPN project which are released under the GPL license (Vranken et al., 2005). The t test for two independent samples was used to assess the significance of differences between mean values. To obtain a measure of TMD hydrophobic lengths the exoplasmic edge was defined as described above for the cytosolic edge and a frequency distribution of resulting TMD lengths determined.

**TMD Asymmetry Analysis**

For each protein in an organelle set the hydrophobic length was defined as above. The “inner leaflet” was defined as the cytosolic edge to the midpoint and the “outer leaflet” the midpoint to the extra-cytosolic edge. The abundance of all residues was normalized for each “leaflet.” For each residue type the abundance in the inner leaflet was then subtracted from the abundance in the outer leaflet, and divided by the total abundance.

**Size Moment**

To test for the presence of flattened interaction faces on TMD helices a “size moment” for the residues along the TMDs was calculated. This is analogous to the hydrophobic moment described by Eisenberg (Eisenberg et al., 1984), and is designed to measure the circular asymmetry of side chain volume around the helix. Residues in a typical α helix are offset by 100°. Thus size moments were calculated by defining each residue as a vector with its volume as its length and its angle as n x 100° (where n = 0 for the first residue, 1 for the second etc). These vectors were then summed over a window of seven residues, i.e., 700° or almost two complete turns of the α helix. This window was then scanned along the TMDs, and moments were plotted with respect to the position of the central residue of the window.

**Artificial Neural Network**

The inputs for the neural network were derived from the residue composition of the sequences in our data sets. However, the data sets each had different numbers of sequences which could bias the network toward the largest input group. Thus sequences were removed at random from all but the smallest data set so that each set consisted of 99 proteins. The amino acid compositions of short stretches of sequence adjacent to and within the TMDs of the proteins in the equilibrated data sets were encoded into numerical vector inputs. The relative abundance of each of the 20 amino acids in a given sequence region corresponded to an input node. The six sequence regions were (−3–0), (1–4), (9–11), (12–15), (16–17), and (18–24) with the cytosolic TMD edge at position zero. This gave 120 input nodes: 6 regions × 20 amino acids.

The neural network was of the feed-forward type with one hidden layer (Me, 2009). Error back-propagation was used to train the neural network. The learning rate was set at 0.01, and there were 100 training cycles. Use of 6 input regions (120 input nodes) and 7 hidden nodes was found to be optimal. For fivefold cross-validation the data sets were randomly partitioned into five subsets, and for each round of testing four subsets were used for training and one was used for testing. Predictive performance was measured using the Matthews correlation coefficient (MCC, (Matthews, 1975)):

$$MCC=\frac{TpTn-FpFn}{\sqrt{\left(Tp+Fn\right)\left(Tp+Fp\right)\left(Tn+Fp\right)\left(Tn+Fn\right)}}.$$

A threshold was set to enable us to classify predictions into true positives (*Tp*), true negatives (*Tn*), false negatives (*Fn*) and false positives (*Fp*). During training this threshold was 0.67. The MCC was used to identify the best neuronal weights to be used in prediction. The MCC is one for a perfect prediction and zero for a random assignment. The MCC, sensitivity and specificity were calculated for all thresholds between zero and one. The mean accuracy of the cross-validation was calculated using the threshold (0.68) with the highest MCC (0.84). The sensitivity was calculated as:

$$Sensitivity=\frac{Tp}{Tp+Fn}.$$

The specificity was calculated as:

$$Specificity=\frac{Tp}{Tp+Fp}.$$

**Subcellular Location Prediction**

The best network from cross-validation testing was trained with the entire size-normalized datasets, and the optimized weights then used to predict the subcellular location of SNARE family proteins, based on their TMD regions. Fungal orthologs of the *S. cerevisiae* SNAREs were obtained by BLAST searching as described above or using the *SNARE* database (Kloepper et al., 2007). To test the topology dependence of the neural network, the SNARE TMD sequences were reversed and treated as type III proteins. In both cases, the highest output score was taken to be the prediction.

To compare the neural network to existing localization predictors, the *S. cerevisiae* proteins from the organelle-specific data sets were tested for predicted localization using the available large-scale predictions and web servers for SherLoc (Shatkay et al., 2007), WoLF PSORT (Horton et al., 2007), or Euk-mPLoc (Chou and Shen, 2007), with the predictors being set to search for fungal localizations where possible. For WoLF PSORT and SherLoc the top prediction was counted, while for Euk-mPloc if the correct localization was present in the prediction it was counted. “Membrane” was assumed to mean plasma membrane. The Euk-mPLoc server only accepts proteins over 50 amino acids long and so some proteins could not be predicted and were not counted. Prediction accuracy was calculated as the percentage of correct predictions out of the whole dataset used for testing. The accuracy of the neural network was calculated by averaging the performances in the rounds of leave-one-out cross-validation.

**Consensus TMDs for Structural Representations**

To represent the differences in the structure of TMDs, “consensus” sequences for fungal organelle sets were generated using the most abundant amino acid at each position in the alignment:

Golgi: RRRRRLLLAALLLLLLLLLSSSSS

Plasma membrane: KKRRRLFFFLILLLLLLVVVVGVVAAIGGSSGS.

Sequences were modeled on an α helix using PyMOL in the surface display mode (DeLano Scientific).

## Supplemental Information

Table S1. Reference Proteins from *S. cerevisiae* Used in the Study, Related to Figure 1 The NCBI RefSeq accession is given, along with the TMD ends as defined using a scanning window, the topology, and the number of orthologs identified prior to redundancy reduction. Relevant literature on localization and topology is collated at the Saccharomyces Genome Database (SGD).

Table S2. Reference Proteins from *H. sapiens* Used in the study, Related to Figure 1 The NCBI RefSeq accession is given, along with the TMD ends as defined using a scanning window, the topology, and the number of orthologs identified prior to redundancy reduction. Relevant literature on localization and topology is collated at the Topology Data Bank of Transmembrane Proteins (TopDB) (Tusnády et al., 2008). Proteins of the apical and basolateral domains of vertebrate epithelial cells were from literature sources (Decaens et al., 2008; Delacour and Jacob, 2006; Mellman and Nelson, 2008).

Table S3. Residue Abundance in the Organelle-Specific TMD Datasets from Fungi, Related to Figure 2 Normalized abundances for each residue at positions through the TMD alignments from the indicated organelle datasets. These numerical values are displayed graphically in Figure 2A.

Table S4. Residue Abundance in the Organelle-Specific TMD Datasets from Vertebrates, Related to Figure 2 Normalized abundances for each residue at positions through the TMD alignments from the indicated organelle datasets. These numerical values are displayed graphically in Figure 2B.

Document S1. Article Plus Supplemental Information
